# Prospects of Using Termite Mound Soil Organic Amendment for Enhancing Soil Nutrition in Southern Africa

**DOI:** 10.3390/plants9050649

**Published:** 2020-05-20

**Authors:** Kafula Chisanga, Ernest R. Mbega, Patrick A. Ndakidemi

**Affiliations:** 1 Department of Sustainable Agriculture, The Nelson Mandela African Institution of Science and Technology, P.O. Box 447, Arusha 23311, Tanzania; ernest.mbega@nm-aist.ac.tz (E.R.M.); patrick.ndakidemi@nm-aist.ac.tz (P.A.N.); 2 Centre for Research, Agriculture Advancement, Teaching Excellence and Sustainability (CREATES) in Food and Nutrition Security, The Nelson Mandela African Institution of Science and Technology, P.O. Box 447, Arusha 23311, Tanzania

**Keywords:** soil conditioner, soil fertility, soil nutrient bioavailability, soil pH, soil physical and chemical properties

## Abstract

Termite mound soils are reportedly utilized as an alternative to NPK fertilizers by cash constrained smallholder farmers in some parts of Southern Africa. However, there is limited knowledge regarding their mineral nutritional value. The intention of this work was therefore to investigate the macro and micronutrient composition of different sections of the termite mounds; top, base and neighboring areas. The study approach involved physical and chemical analysis of 36 sites across Pemba and Choma districts in Southern Zambia through collection of soil samples in triplicate at 0–20 cm depth, using a soil auger. Findings revealed that the soil pH had elevated levels in the base segments of the termite mounds compared with the top and the neighbouring soils. However, elevated N, P and K levels were recorded in the top sections with significant differences (*p* < 0.05) in clay and silt composition observed. Additionally, metallic micronutrients, Cu and Zn were also found to be elevated in termite mounds in contrast to surrounding soils. We concluded that top termite mound soil should be considered as part of an integrated nutrient management strategy by financially challenged smallholder farmers cultivating in light textured soils of southern Africa.

## 1. Introduction

The Food and Agriculture Organization of the United Nations reports that soil fertility management practices are a major barrier to agricultural production in developing countries [1]. This is further exacerbated by high input costs especially mineral fertilizer which is a critical ingredient in crop production especially for the principal cash and basic crops like maize. Umar et al. [2] also affirms that declining soil fertility in sub-Saharan Africa (SSA) continues to reduce soil productivity and is a major obstacle to addressing problems of food security. In Zambia, for instance, cash strapped smallholder farmers in some agriculture productive districts of Pemba and Choma, in the southern part of the country have often used termite mound soil to fertilize their crops to cushion against the inorganic fertilizer costs [3]. 

These districts are major contributors to overall crop production in Southern Zambia whose major crops grown by smallholder farmers include maize, cotton, sunflower, sorghum, cowpeas, groundnuts, beans and soybeans [4]. Most of the soils in the area are sandy and pose a great challenge to poor smallholder farmers who have limited financial resources to purchase commercially available fertilizer. Such degraded soils have a great impact on the livelihood tactics of the resource constrained smallholder farmers [5]. 

In Southern Zambia, termite mounds are abundant in Pemba and Choma districts and smallholder farmers have always resorted to using them as a source of alternative fertilizer for crop production [3,6,7]. Ruiz-Diez et al. [8] revealed that deficiency in nutrients along with erratic rainfall hold back plant growth. Nziguheba et al. [9] also confirmed that SSA has been well-known for nutrient mining attributable to limited utilization of commercially available fertilizer by some smallholder farmers. Because of this factor, phosphorus deficiency is a key challenge to agriculture production in many countries of SSA [10]. Imbalance of elements such as P affects nodule formation for the legume crops establishment including N_2_ fixation and plant matter production [11]. We argue here that having information on the nutrient status of the termite mound is crucial for advising on the sections which could be appropriate to use as soil amendment material in agricultural production and as a component of integrated nutrient management (INM) system at smallholder farmer level especially in the developing world. From the point of view of soil science, termite mounds are rich in macro and micro nutrients due to the termite bioturbation activities that enhances significantly exchangeable cations (K, Ca, Mg), organic matter, soil pH and micro nutrients such as Fe, Zn and Cu resulting from the material they collect, ingest and excrete [12]. 

Until today, studies conducted in Zambia have concentrated on general termite mound soil characterization with little attention on the status of the part of the termite mounds that provide better nutrients for crop production. The current practice by the cash constrained smallholder farmers has been collecting soils from anywhere around the termite mound and applying it directly on to the fields, either solely or in combination with cattle manure and commercially available fertilizer [6]. The smallholder farmers have limited knowledge as to which sections of the termite mounds holds’ better fertility. Thus gaining knowledge on the best collection areas could be a game changer in providing the much needed N and P nutrients for improved crop production and productivity for future integrated soil fertility management (ISFM) options at smallholder farm level in southern Africa. 

Our work, therefore, attempts to discuss the unknown fertility status potentials of the top or base termite mound soil used in agricultural production by financially challenged smallholder farmers in Southern Zambia as part of a traditional integrated nutrient management strategy. In this regard, the objective of the current study was to evaluate soil properties and illustrate differences in their composition, both chemical and physical arising from the top sections of the termite mound to the basement and into the neighbouring areas. We envisage that for the future, the information generated could be useful in soil fertility management decisions for agricultural production, from the smallholder farmer perspective located in similar environments in southern Africa. Accordingly, we hypothesized that different sections of termite mounds have varying nutrient levels and thus may have auxiliary effects when applied as a soil amendment in soils lacking organic matter or light textured soils for boosting plant growth.

## 2. Materials and Methods 

### 2.1. Study Site

**Figure 1:**
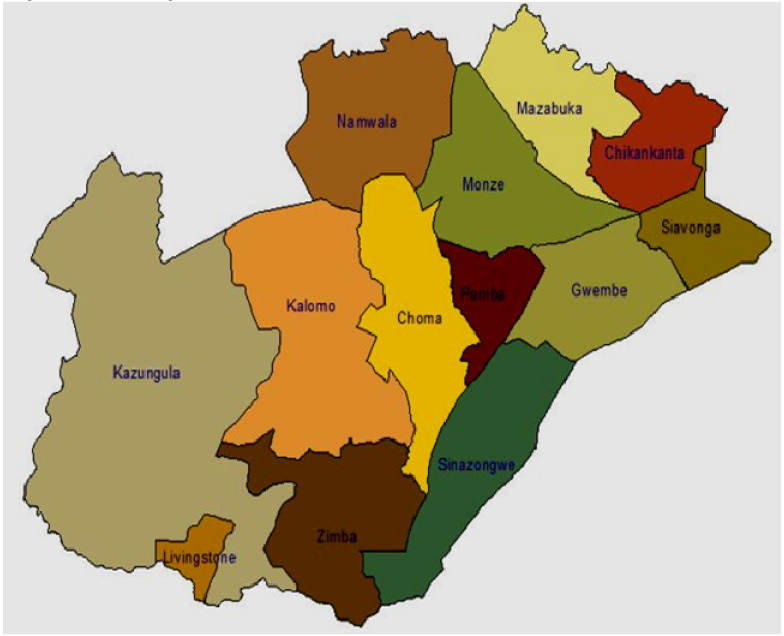
Study location—Choma and Pemba districts in Southern Zambia. (Source: Ministry of Agriculture, Zambia).

The study was conducted in southern province of Zambia in Pemba (16° 32’ 0” South, 27° 22’ 0” East) and Choma (Longitudes 26° 30′ 0” and 27° 30′ 0” East of Greenwich and Latitudes 16° and 17° 45′ 0” South). Pemba and Choma districts (Figure 1) fall in Agro Ecological Zone (AEZ) IIa of the country where maximum rainfall ranges from 800–1000 mm yr^−1^ and the dominant soils are the Lixisols, Regosols, Leptosols and Vertisols [4]. The Pemba climate is classified as Cwa by the Köppen–Geiger syste—a zone of climate characterized by warm and temperate conditions. In comparison to winter, the summers have much more rainfall. Mean annual temperature is 19.5 °C while mean annual precipitation is 848 mm. The driest month is July, with 0 mm of rainfall. In December, the precipitation reaches its climax, with an average of 219 mm. The warmest month of the year is November, with a mean temperature of 23.1 °C and the coldest month is July (14.0 °C). Just like Pemba, the climate in Choma is classified as Cwa following the Köppen–Geiger system coupled with warm weather scenarios. In winter, there is much less rainfall than in summer. Annual mean temperature is 18.7 °C while that of rainfall is 805 mm, with July being the driest (0 mm of rain). Most precipitation falls in January (202 mm). The warmest month of the year is November (22.4 °C). In July, the mean temperature is 12.9 °C and is considered the lowest of the whole year. The principal vegetation in southern province of Zambia is the Kalahari woodlands, characterized by *Mopane* with patches of miombo woodland, *acacia species* and termite mounds [13]. Elevation range of the undulating surface is from 1200–1350 m above sea level [14]. 

### 2.2. Termite Mound Sampling Design

**Figure 2:**
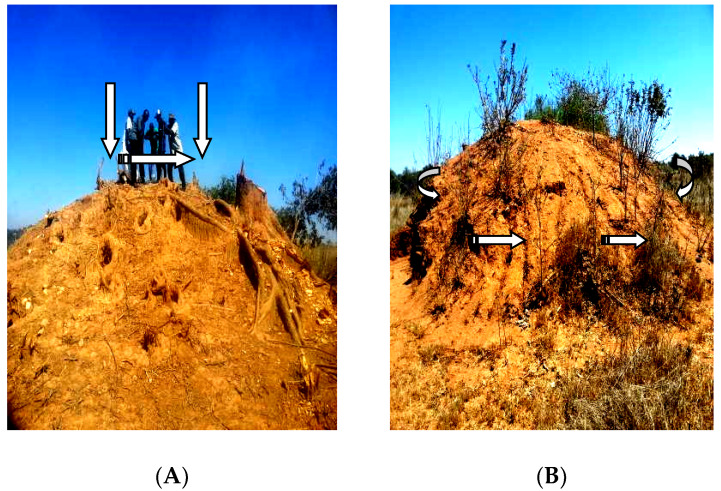
Photographs of termite mound soil sampling points: (**A**) Soil collection from the top of the termite mound; (**B**) soil collection along the base of the termite mound.

Prior to collection of soil samples from termite mounds, a field research permit was obtained from the IRB (Approval No. 2018—Feb—046). Subsequently, a rapid assessment of the study areas was undertaken during the cropping season to identify the termite mounds which were in use for crop production by the smallholder farmers. Six plots measuring 500 × 20 m were marked out in each study district, with corners set and bench marked using wooden pegs. All the termite mounds in the area both used and non-used were counted and two termite mounds were selected at random for sampling [15]. Soil samples (n = 36) were taken at the depth of 0–20 cm from three different points; top, base and 10 m away from the centre of the termite mounds using a soil auger [16] (Figure 2). A composite sample made up of three subsamples was collected. Thereafter, a representative sample of about 1 kg of soil was packed in a plastic, labelled and taken to laboratory. All the collected soil samples were air dried and passed through a 2 mm-sieve. 

### 2.3. Laboratory Analytics

The study adopted the standard methods of soil analysis for the parameters of interest as indicated in Table 1. All the laboratory measurements were conducted in triplicate.

### 2.4. Statistical Analysis

A two–way analysis of variance (ANOVA) using randomised complete block design approach with STATISTICA Version 10 Programme (2011) was employed. Mean significant differences were tested at 95% confidence interval. The normality and homogeneity of variances was met before the ANOVA (Shapiro–Wilk test). Correlation analysis was further employed to locate the relationship between organic carbon and other available mineral elements (N, P, K, Cu, Fe and Zn). The study adopted the factor effects following Wang and DeVogel [23] statistical model for data analysis and was applied as specified below:

*Y_ijk_ = µ+* α *_i_ + β_j_ + (*α*β) _i.j_ +* ε*_ijk_* for i = 1, 2...b; j = 1, 2...t; k = 1, . . . , nij

where *Y_ijk_* = observations; *µ* = grand mean; α*_i_* = main effects of factor A; *β_j_* = main effects of factor B; *(*α*β) _i.j_ =* interaction effects between factor A and B; ε*_ijk_* =, error term. The site (district) and the soil collection points (top, base and 10 m away from termite mound) were used as predictor variables instead of pH; N; P; K; Ca; Mg; O.C; CEC; EC; Zn; Cu; Fe and clay, silt and sand that represented response variables.

## 3. Results

### 3.1. Soil pH

The soil pH in the top termite mound averaged 6.53 while that of the base termite mound 7.05 and in soil samples from 10 m away from the termite mound pH values averaged 5.08. Generally, Pemba had higher pH values than Choma site. 

### 3.2. Total N

Total N in the top termite mound soil averaged 0.08% while base termite mound soil and that collected 10 m away from it indicated an average of 0.07% and 0.06% respectively. The proposed threshold level for N was 0.20% [24]. Soil having critical levels below the suggested value is considered deficient in plant available N (Table 2). 

### 3.3. Available P

The available P in the top termite mound soil averaged 8.04 mg kg^−1^ across the two study districts. For the base termite mound soil, P values averaged 6.34 mg kg^−1^, while in the surrounding soil, 10 m away from it, the average value was 2.30 mg kg^−1^. Overall, Pemba District exhibited highest P content in the top and base termite mound sections including 10 m away from the structure compared with Choma (Table 2). This was however below the threshold levels for P pegged at 15 mg kg^−1^. 

### 3.4. Exchangeable Mg and K

Exchangeable Mg averaged 3.33 cmol kg^−1^. The proposed threshold value in most agriculture crops was 0.2 cmol kg^−1^ [24]. In the top termite mound soil, Mg averaged 4.97 cmol kg^−1^, suggesting that Mg supply was adequate to support crop growth. For the base termite mound soil, the Mg average level was 4.57 cmol kg^−1^ while values for soil collected 10 m away from it, the Mg values averaged 0.44 cmol kg^−1^.

Exchangeable K in the top termite mound soil averaged 89.85 cmol kg^−1^ while for the base termite mound soil, the average value was 23.91 cmol kg^−1^ whereas the soil collected 10 m away from the termite mounds, exhibited an average of 0.17 cmol kg^−1^ (Table 2). Termite mound soil had K levels above the critical level of 2 cmol kg^−1^.

### 3.5. Exchangeable Ca

Exchangeable Ca averaged 52.58 cmol kg^−1^ in the top termite mound soil of the study sites. Base termite mound soil exhibited an average level of 30.53 cmol kg^−1^ while the soil 10 m away from the structure yielded an average of 3.09 cmol kg^−1^ (Table 2). Recommended threshold for Ca is 5 cmol kg^−1^.

### 3.6. Organic Carbon (OC)

OC averaged 1.31% in the top termite mound soil (Table 3). In the base termite mound soil, the average was 0.97% whereas in the soil collected 10 m away from the structure, the average was 0.83%. 

### 3.7. Cation Exchange Capacity (CEC)

CEC averaged 32.19 cmol kg^−1^ in the top termite mound soil in both study sites (Table 3). Base termite mound soil exhibited CEC average of 30.09 cmol kg^−1^ whereas soil 10 m away from it, had an average of 4.21 cmol kg^−1^. Most sites had CEC above critical value except two sites away from the termite mounds in Pemba and Choma districts which were below 10.0 cmol kg^−1^, the recommended minimum level. 

### 3.8. Electrical Conductivity (EC)

EC values averaged 439.99 µs cm^−1^ in the top termite mound soil whereupon in the base termite mound soil the average was 904.48 µs cm^−1^. For soils collected 10 m away from the termite mound, the EC average was 54.20 µs cm^−1^. Generally, the termite mound soil had higher values of EC compared with the surrounding soil across the study districts (Table 3) but adequate to support crop growth. 

### 3.9. Available Cu, Fe and Zn

Available Cu in the top termite mound soil averaged 0.88 mg kg^−1^. For base termite mound soil, the average was 1.19 mg kg^−1^ while the soil collected 10 m away from it exhibited an average of 0.13 mg kg^−1^. All the soils from the termite mounds had greater than threshold value of 0.2 mg kg^−1^ except for sites in soil collected 10 m away from the termite mound (Table 3). Available Fe in the top termite mound soil averaged 25.84 mg kg^−1^. In the base termite mound soil, the average was 17.63 mg kg^−1^, whereas in the soil 10 m away from it, Fe content averaged 27.35 mg kg^−1^. Recommended threshold level for Fe ranges from 0.3 to 10 mg kg^−1^ [25]. In all the studied soils, the Fe levels were greater than the critical level. Available Zn in the top termite mound soil averaged 1.45 mg kg^−1^. The Zn levels in base termite mound averaged 1.12 mg kg^−1^ while for soil 10 m away from it, the average was 0.36 mg kg^−1^. For Zn (DTPA), the proposed threshold levels was 0.4–0.6 mg kg^−1^ and any values above 10–20 mg kg^−1^ were considered as having excess Zn levels [26]. All the top and base termite mound soils had adequate Zn levels while soils collected 10 m away from it exhibited Zn deficiency.

### 3.10. Soil Texture

Particle size distribution (PSD) revealed different composition of sand, clay and silt. Clay on top termite mound > clay on base termite mound > clay 10 m away from the termite mound (Table 4). Additionally, there were significant differences (*p* < 0.05) in terms of clay content from different termite mound sections. The mean clay content across the districts was 30.4%. 

For silt composition in this study, the average values were; 5.49%, 11.36% and 6.13% respectively for the top, base and soil collected 10 m away from the termite mound with significant differences (*p* < 0.05) being recorded across the collection areas. The average value between districts was 7.66%. Sand levels were higher in the soil collected 10 m away from the termite mound (62.51%), followed by base termite mound (41.34%) and top termite mound (27.30%) respectively. The mean value across the districts was 43.7% and differences between districts were non- significant (*p* > 0.05). However, significant differences (*p* < 0.05) were observed within the soil collection areas (Table 4).

### 3.11. Relationship between O.C and N, P, K, Cu, Zn and Fe

There was a significant (*p* < 0.05) positive correlation between O.C and N, P, K and Zn. As for Cu and Fe however, there was no significant correlation with O.C. (Table 5). This may be attributable to the higher amounts of bioturbation activities by mound building termites.

## 4. Discussion

Our study showed that the soil pH values in the different soil collection points were alkaline to moderate levels and could support crop growth. However, a pH value of 4.3 could be detrimental to crop growth and would call for lime application to raise pH to acceptable levels for crop production. Chapoto et al. [4] reported that acceptable pH levels for most crops are 5.5–5.8 and at these scales there is no advantage from liming. In addition, Fairhurst [24] reported that P availability is greatest at pH 5.5–7.0. Furthermore, soil organisms required for N mineralization function best at soil pH 5.5–6.5 and at this level, all micronutrients, except Mo, are more available from pH 5.5 to 6.0. Mn and Fe toxicity is also drastically reduced in this range. 

Though slightly higher average percentage of total N was found in the top termite mound soil compared with base termite mound soil and soil collected 10 m away from it, this was however marginally below the proposed critical threshold. Sarcinelli et al. [16] reported that higher values of N in termite mounds may be attributed to fine organic material (twigs, grass etc.) resulting from nest building activities of the ants. In any case, if the smallholder farmers in Pemba and Choma study districts of Southern Zambia with little capacity to purchase inorganic fertilizer continue using the termite mound soil as an amendment, it would be more beneficial for them to enhance N levels through application of top and/or base termite mound soil combined with other organic resources such as cattle manure and implementation of crop rotation with legume crops that may involve common beans, soybeans, groundnuts, cowpeas, pigeon peas etc. In such a situation, warranted corrective measures for enhanced N levels would include application of top or base termite mound soil as there were no significant differences (*p* > 0.05) between the two sections with regard to N content. However, for smallholders who would afford commercially available fertilizers, combining with termite mound soil may be the best strategy for boosting N availability. Studies conducted by Mtambanengwe et al. [27] in Zimbabwe reported that combination of N mineral fertilizers and organic resources increased the organic matter loading in the soil which often resulted in farmers achieving high crop yields on coarse sandy soils. Ndakidemi and Semoka [28] in a similar study, in Tanzania, recommended application of organic and/or inorganic fertilizers where N levels were below suggested critical levels.

Availability of P is essential for controlling crop growth and development [29]. Overall, in this study, Pemba district exhibited highest P content in the top and base termite mound sections including 10 m away from the structure compared with Choma. However, this was not significant (*p* > 0.05). With this scenario, it would be beneficial for resource constrained smallholder farmers to collect termite mound from the top unlike the base for application in their agriculture fields as there is relatively higher P content. This may be supplemented with cattle manure to enhance the organic matter content of the soil and other limited nutrients. Hernandez et al. [30] reported that feeding and the manner of construction influenced the P content of termite mounds. In the current study however, soils collected 10 m away from the termite mound had the least concentration of P, attributed to the inherent parent material. Generally, P is one of the limiting nutrients in Southern Zambia. A study by Yerokun [31] also indicated that soils of different origins within the country showed similar lower trends in their amount of available P. 

All the studied sites had sufficient Mg levels, except a few in Choma district, suggesting that to attain optimum crop yield, application of termite mound soil, cattle manure and commercially available fertilizers would provide supplemental Mg.

For most crops grown in southern Africa, Fairhurst [24] recommended 0.2 cmol kg^−1^ as a critical level of exchangeable K in soils. Nearly all the soils collected from top and base termite mound had K levels above critical values compared with those collected 10 m away from it. One site in Choma district had K levels below critical value implying that to enhance the K levels in the deficient area there was need to apply termite mound soil, cattle manure and commercially available fertilizers as supplement for K.

The proposed critical level of Ca for majority of crops was 0.5 cmol kg^−1^ [24]. All the sites had Ca levels above the suggested critical value, a situation that implied that the cation was the most prominent on the soil colloids. Additionally, Pemba district exhibited highest Ca content in the top termite mound soils in comparison with Choma. Kristiansen et al. [32] observed that termite mounds are enriched with inorganic elements such as Ca, compared with nearby surface soils resulting from the termite mound building ants’ collection of various woody debris and foraging activities. 

The critical threshold of SOC is pegged at 0.4% [22]. Anything below this poses a critical loss in soil health which may not support proper crop growth. In this respect, Sarcinelli et al. [16] indicated that higher values of SOC observed in the top termite mounds are attributable to termite action of swallowing soil organic matter which is returned as faecal pellets. Soil organic carbon is a measure of the readily available oxidizable content of organic matter, which directly influences nitrogen supplying capacity of the soil. 

Minasny and Mcbratney [33] asserted that a 1% mass increase in soil organic carbon (or 10 g C kg^−1^ soil mineral), based on average, increased water content at saturation, field capacity, wilting point and available water capacity by: 2.95, 1.61, 0.17 and 1.16 mm H_2_O 100 mm soil^−1^, respectively. The increase is reported to be in the order; sandy soils > loams > clays. Chapoto et al. [4] were of the opinion that most of the agricultural lands across southern Africa including Zambia, lacked the much-required organic matter, which is cardinal to the fertility of soils. Absence of required organic matter has negative consequence on the physical, chemical and microbial health of soils. Mtangadura et al. [34] reported that organic nutrient resources which are accessible by smallholder farmers in southern Africa have great potential to enhance soil organic matter despite having differences in their chemical quality and mineral N fertilization regimes.

CEC averaged 32.2 cmol kg^−1^ in the top termite mound soil in both studied sites. Fairhurst [24] grouped soils having <10 cmol kg^−1^ as inadequate in exchangeable bases. Base termite mound soil exhibited CEC average of 30.09 cmol kg^−1^ whereas soil collected 10 m away from the structure had an average of 4.21 cmol kg^−1^. Most sites had CEC above critical value except for two sites away from the termite mounds in Pemba and Choma districts which were below 10.0 cmol kg^−1^, the recommended minimum level. Such soils indicated that they have low cation retention capacity for Ca^2+^, K^2+^ and Mg^2+^. In order to correct the situation, application of organic fertilizers would suffice as they contained high soil organic matter, which is an important source of cation exchange capacity.

The results showed that the termite mound soil had higher values of EC compared with the adjacent soil across the study districts. Knowledge of EC of the soil is important as it renders information about the concentration of soluble salts in the soils. Raina et al. [22] reported that crops like maize, wheat, sorghum and rice among others are medium salt tolerant crops. It is, therefore, a must to have an idea of the salts in a particular soil in order to know its suitability for different crops. Soils with less than 2000 µs cm^−1^ EC values are considered to have low salinity. 

Fageria and Baligar [12] reported that mound termite activities have a significant direct influence on the soil chemical properties which enhances their fertility. Lindsay and Cox [25] proposed critical level for different crops ranging from 0.3 to 10 mg kg^−1^. In all the studied soils, the Fe levels were above the critical level.

Both the top and base termite mound soils had adequate Zn levels while soils collected 10 m away from the structure exhibited Zn deficiency. A study conducted by Manzeke et al. [35] in Zimbabwe indicated that inadequate Zn levels threatens crop production and food nutrition in most cereal-based cropping systems across Africa, a scenario that was also observed in the studied sites. Steffan et al. [36] stressed that the state of a soil has implications on a particular soil’s capability to make available services for growing nutritious foods. 

Generally, the relationship between organic carbon and some available mineral elements in various soil collection points (n) differed. This may be attributable to the termite mound building activities’ that influence the nutrient cycling regimes in the soil environment [12] (Table 4). 

The amounts of clay fractions found in various termite mound sections were similar in magnitude to those observed by other scientists. For instance, a study by Haitao et al. [37], in China, found that the silt and clay content of termite mounds were higher than for adjacent non-termite mound soil. In this regard, ants which are found in termite mounds are responsible for affecting the soil physical characteristics through their activities whereby the small particles are moved from the deeper layers to the surface, burying the organic matter deeper, resulting in changes in soil particle constituents.

**Figure 3:**
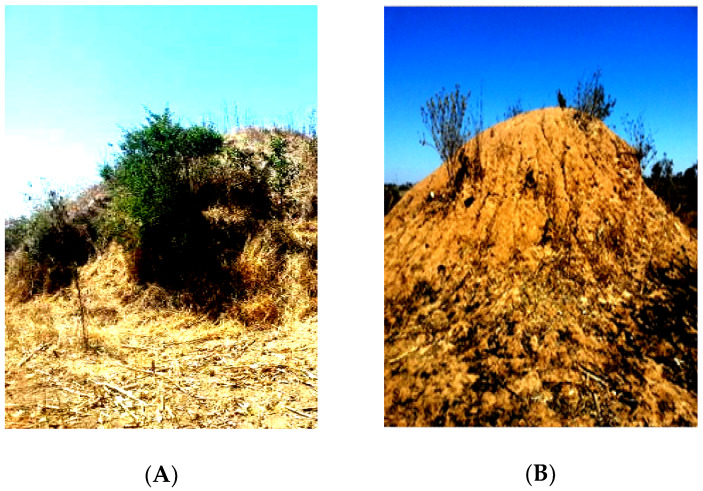
(**A**) Termite mound with vegetation cover is an indicator of good fertility; (**B**) termite mound with no or little vegetation cover depicts poor fertility and is never utilized for agricultural production as part of low input nutrient management strategy by smallholder farmers in Southern Zambia. Indigenous knowledge is normally applied by the mound soil users to identify suitability in crop production [7].

**Figure 4:**
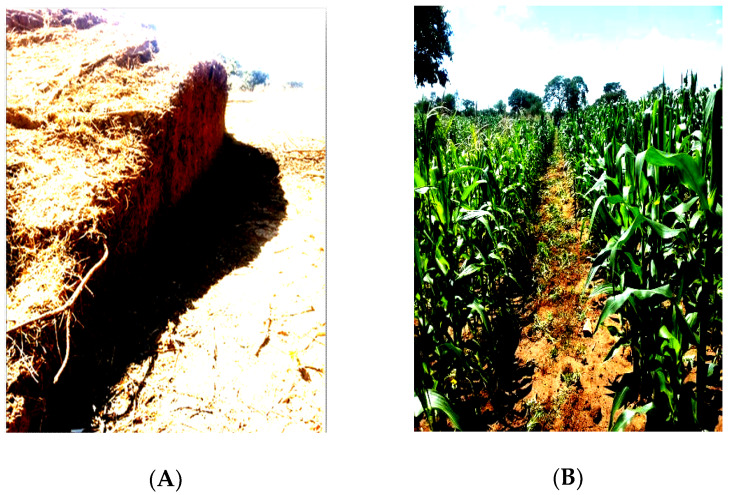
(**A**) Part of cut termite mound soil applied for fertility improvement; (**B**) Maize crop under termite mound soil treatment as part of integrated nutrient management in a conservation agricultural system, Southern Zambia.

These observed differences in textural classes were a result of the original nature of the parent material in studied sites that was mainly composed of sandy. For instance, in a study conducted by Chapoto et al. [4] it was indicated that most of the soils in Agro Ecological Zone IIa, in which the studied areas are located, are of sandy texture. The higher sand percentage in the studied sites gave an indication that the soils were infertile and low in major crop nutrients such as N, P, K and SOC. This conforms with a study conducted by Wyngaard et al. [29] who revealed that most Zambian agricultural soils had inherent small amounts of P in them. The low levels of P availability were linked to the low organic matter composition, nature of the soil, exacerbated by the microenvironments where they were found (Figure 3 and Figure 4).

## 5. Conclusions

The study demonstrated that soil from the top and base sections of the termite mound structures in Southern Zambia, contain potential macro and micro nutrients (P, K, Ca, Mg, Cu, Fe and Zn) which can substantially support crop growth. This can play a key role in integrated nutrient management systems, where manure, crop rotation, intercropping or application of commercially available fertilizer may be used to boost N and P levels in soils lacking these nutrients.

Top sections of the termite mounds exhibited more fertility than the base and the neighbouring soils. This may be attributed to the foraging activities of ants responsible for building termite mounds. During formation, the ants collect organic materials from the surrounding areas and use it for constructing the termite mounds thereby enriching them with nutrients.

In view of the above, we recommend that future studies should focus on growing crops using soil collected from different sections, top and base of the termite mounds and establish the performance under such soil amendment conditions. There is also a need to determine at least optimal rates of termite mound soil for application in agricultural fields especially under conservation agricultural systems to boost crop production and productivity in combination with organic and commercially available mineral fertilizer. 

## Figures and Tables

**Table 1:** Summary of methods used for soil analysis.

Nutrient Variable	Units of Measurement	Method Applied	References
pH (water)	pH	1:1 (soil:H_2_O)	VanReeuwijk [17]
Organic Carbon	%	Walkley and Black	Walkley and Black [18]
Total N	%	Kjeldahl method	Page [19]
Available P	mg/kg	Bray II (molybdate blue) method, spectrophotometer	Bray and Kurtz [20]
Exchangeable K	mg/kg	1 M NH_4_Cl, flame photometer	VanReeuwijk [21]
Exchangeable Ca	cmol/kg	1 M NH_4_Cl, flame photometer	VanReeuwijk [21]
Exchangeable Mg, Cu, Fe, Zn	cmol/kg mg/kg	1 M NH_4_Cl, atomic absorption spectrophotometer	VanReeuwijk [21]
Electrical Conductivity		Conductivity meter procedure	Raina et al. [22]
Cation exchange capacity (CEC)	cmol/kg	Exchangeable K + Na + Ca + Mg + Al + H	Page [19]
Sand	%	Hydrometer method	VanReeuwijk [21]
Silt	%	Hydrometer method	VanReeuwijk [21]
Clay	%	Hydrometer method	VanReeuwijk [21]

**Table 2:** Comparison of soil chemical parameter values and treatment effects across studied sites.

**District**	**pH**		**N (%)**	**P (mg kg^−1^)**	**K (cmol kg^−1^)**	**Ca (cmol kg^−1^)**	**Mg (cmol kg^−1^)**
Pemba	6.59 ± 0.29a		0.09 ± 0.01a	6.12 ± 1.11a	1.53 ± 0.31b	44.91 ± 13.82a	5.64 ± 1.36a
Choma	5.85 ± 0.30b		0.05 ± 0.00b	4.99 ± 0.72a	74.47 ± 31.53a	12.55 ± 2.93b	1.01 ± 0.17b
SCA							
T	6.53 ± 0.34b		0.08 ± 0.01a	8.04 ± 0.94b	89.85 ± 46.32a	52.58 ± 19.33a	4.97 ± 1.61b
B	7.05 ± 0.19b		0.07 ± 0.01a	6.34 ± 1.24b	23.91 ± 11.42ab	30.53 ± 6.90ab	4.57 ± 1.45b
A	5.08 ± 0.31a		0.06 ± 0.01a	2.30 ± 0.42a	0.17 ± 0.05b	3.09 ± 0.85b	0.44 ± 0.09a
**Overall Treatment Effects–F statistics**							
District		0.03 *	0.00 *	0.31	0.01 *	0.01 *	0.00 *
SCA		0.00 *	0.26	0.00 *	0.03 *	0.01 *	0.00 *
District * SCA		0.6	0.85	0.95	0.03 *	0.03 *	0.05

**NB:** * Significant at *p* ≤ 0.05; SCA = soil collection area (n = 36), while A, B and T means soil collected 10 m away, base and top sections of termite mound respectively. Means within the same column followed by the same letter (s) refer to no significance at (*p* ≤ 0.05) based on Tukey’s Honest Significance Difference Test.

**Table 3:** Comparison of soil chemical parameter values and treatment effects across studied sites.

**District**	**O.C (%)**	**CEC (cmol kg^−1^)**	**EC (µs cm^−1^)**	**Zn (mg kg^−1^)**	**Cu (mg kg^−1^)**	**Fe (mg kg^−1^)**
Pemba	1.01 ± 0.05a	30.14 ± 7.67a	841.65 ± 262.36a	0.91 ± 0.19a	0.81 ± 0.16a	18.02 ± 2.37b
Choma	1.07 ± 0.06a	14.18 ± 2.92b	90.81 ± 18.46b	1.05 ± 0.16a	0.66 ± 0.13a	29.19 ± 1.18a
SCA						
T	1.31 ± 0.02a	32.19 ± 8.92b	439.99 ± 111.27ab	1.45 ± 0.21b	0.88 ± 0.20b	25.84 ± 3.51b
B	0.97 ± 0.04b	30.09 ± 6.98b	904.48 ± 391.56a	1.12 ± 0.21b	1.19 ± 0.08b	17.63 ± 2.59a
A	0.83 ± 0.04c	4.21 ± 1.06a	54.20 ± 11.96c	0.36 ± 0.04a	0.13 ± 0.05a	27.35 ± 0.88b
**Overall Treatment Effects—F Statistics**						
District	0.12	0.03 *	0.00 *	0.44	0.31	0.00 *
SCA	0.00 *	0.00 *	0.01 *	0.00 *	0.00 *	0.00 *
District * SCA	0.04 *	0.25	0.01 *	0.01 *	0.93	0.03 *

**NB:** * Significant at *p* ≤ 0.05; SCA = soil collection area (n = 36), while A, B and T means soil collected 10 m away, base and top sections of termite mound respectively. Means within the same column followed by the same letter (s) refer to no significance at (*p* ≤ 0.05) based on Tukey’s Honest Significance Difference Test.

**Table 4:** Comparison of soil physical parameter values and treatment effects across studied sites.

**District**	**Clay (%)**	**Silt (%)**	**Sand (%)**
Pemba	33.41 ± 6.27a	7.66 ± 1.33a	41.58 ± 7.04a
Choma	27.30 ± 6.26a	7.65 ± 1.16a	45.85 ± 7.56a
SCA			
T	56.62 ± 7.06a	5.49 ± 1.81b	27.30 ± 6.15b
B	29.98 ± 2.54b	11.36 ± 1.25a	41.34 ± 6.59ab
A	4.47 ± 2.17c	6.13 ± 0.69b	62.51 ± 10.50a
**Overall Treatment Effects—F Statistics**			
District	0.26	0.99	0.67
SCA	0.00 *	0.01 *	0.02 *
District * SCA	0.83	0.08	0.93

**NB:** * Significant at *p* ≤ 0.05; SCA = soil collection area (n = 36), while A, B and T means soil collected 10 m away, base and top sections of termite mound respectively. Means within the same column followed by the same letter (s) refer to no significance at (*p* ≤ 0.05) based on Tukey’s Honest Significance Difference Test.

**Table 5:** Correlation matrix table between organic carbon and key macro and micronutrients in the studied areas.

Parameter	N	P	K	Cu	Zn	Fe
O.C	0.34 *	0.48 *	0.45 *	0.33ns	0.51 *	0.04ns

* Correlations are significant at *p* ≤ 0.05; ns = non significant.
